# Comprehensive Characterization of Conformational Dynamics in Risk and Resilience Alleles of Apolipoprotein E for Alzheimer’s Disease using Long‐Time Scale Molecular Simulations And Graph Neural Networks

**DOI:** 10.1002/alz.089342

**Published:** 2025-01-03

**Authors:** William Martin, Feixiong Cheng

**Affiliations:** ^1^ Cleveland Clinic, Cleveland, OH USA; ^2^ Case Western Reserve University, Cleveland, OH USA

## Abstract

**Background:**

Apolipoprotein E (ApoE) is the primary cholesterol and lipid transporting apolipoprotein in the central nervous system (CNS) and is the greatest genetic risk factor for Alzheimer’s Disease (AD). There are three main isoforms differing by single amino acid changes: ε3 is “neutral”, ε4 is “risk” (Cys112Arg), and ε2 is “resilience” (Arg158Cys). Rare forms (Christchurch, Jacksonville) have also been proposed as resilience alleles, while an ε4‐like allele (with Arg61Thr) is present in non‐human primates without AD risk. To date, only a single mutated NMR structure of full‐length ε3 in a closed conformation exists, leaving unanswered questions regarding conformational differences between isoforms. Additionally, nearly all experimental data for ApoE considers multimeric structures as opposed to the biologically relevant monomer.

**Method:**

Here, we have integrated a graph representation of long‐time scale molecular simulation data with neural networks to generate reversible Markov state models. Our method utilizes multiple replicates of long‐timescale (six replicates of 15 µs per isoform, 540µs in total) to generate 200 starting conformations per isoform, each of which was simulated in triplicate for and additional 1 microsecond (600 µs per isoform). In total, 4.14 milliseconds of simulation across 6 isoforms were generated. We use Markov state models generated using GraphVAMPNets (a deep neural network utilizing graphs as the input data) in conjunction with DeepMSM, elucidating key macrostates for each isoform which may dictate the differences in conformation, informing the risk versus resilience potential. Finally, we integrated CoVAMPNet, a framework for directly comparing macrostates from different datasets.

**Result:**

We have identified conformational differences between comparable macrostates which may inform differences in risk or resilience across the isoforms. This elucidation is a result of our integration of protein energy networks with molecular modeling, and may inform the development of future therapeutics targeting ApoE in AD. Mapping of macrostates to each other and comparing mean first passage times indicates potential risk and resilience states, with ε4 having lower transition times into a specific state and ε2 having a particularly stable state.

**Conclusion:**

We have identified putative risk or resilience conformational states in APOE, which may have implications for therapeutic discovery.

